# Analysis of Micro-Evolution Mechanism of 3D Crack Initiation in Brittle Materials with Hole under Uniaxial Compression

**DOI:** 10.3390/ma17040920

**Published:** 2024-02-16

**Authors:** Semaierjiang Maimaitiyusupu, Zhende Zhu, Xuhua Ren, Hui Zhang, Shu Zhu

**Affiliations:** 1 College of Water Conservancy & Hydropower Engineering, Hohai University, Nanjing 210024, China; renxh@hhu.edu.cn (X.R.); zhangh1998hhu@163.com (H.Z.); 2 College of Civil Engineering, Kashi University, Kashgar 844006, China; 3 Jiangsu Research Center for Geotechnical Engineering Technology, Hohai University, Nanjing 210098, China; zzdnj@hhu.edu.cn (Z.Z.); 20210940@hhu.edu.cn (S.Z.); 4 Key Laboratory of Ministry of Education for Geomechanics and Embankment Engineering, Hohai University, Nanjing 210098, China

**Keywords:** transparent brittle materials, pre-existing cracks, hole, uniaxial compression

## Abstract

This article investigates the microscopic mechanism of crack initiation and propagation in three-dimensional embedded cracks in brittle materials containing circular holes. First, a method for the development of transparent, brittle materials is proposed. Second, UCS tests were conducted on transparent, brittle materials containing circular holes and internally embedded three-dimensional cracks. Finally, a numerical model was established in PFC3D to analyze the crack initiation and propagation mechanism. The results show that when α = 0° (α refers to the pre-existing crack inclination), the upper tip of the pre-existing crack appears as a tensile wing crack, and the lower tip of the pre-existing crack appears as a tensile–shear mixed crack. When α = 30°, no wing crack appears, and the tensile crack on the fracture surface only appears after the hole cracks. When α = 60 and 90°, a tensile wing crack and an anti-wing tensile–shear mixed crack appear at the upper tip of the pre-existing crack. A tensile wing crack appears at the lower tip of the pre-existing crack and appears “self-limiting”. During the propagation of wing cracks to the surface of the specimen, the transition sequence of the crack propagation mechanism is tensile through failure—tension-shear mixed failure—tensile failure. It can be seen that the interaction between the crack and hole has an important influence on the evolution mechanism of the crack and the failure mode of the specimen.

## 1. Introduction

The internal primary cracks in rock masses have a very important influence on the strength and stability of the rock mass. Many instability and collapse accidents in rock masses are the result of the propagation and eventual penetration of internal primary cracks under external forces. In response to this issue, previous researchers have conducted a large number of experimental studies to investigate the propagation and penetration of internal cracks in rock masses under compression. However, due to the limitations of experimental conditions, most previous studies were focused on the propagation and penetration of two-dimensional cracks or through-going cracks. As the actual compression of rock masses is a three-dimensional problem, studying the three-dimensional mechanism of crack initiation and propagation will have significant theoretical and practical implications for understanding the propagation of internal cracks in real rock masses.

Many researchers have studied the effects of crack shape, size, position, and inclination angle on the initiation, propagation, and failure processes of pre-existing cracks using different materials. For example, Soheil Abharian et al. [1] studied the mechanical behavior of gypsum specimens with linearly arranged round holes at different angles and notches of different sizes under uniaxial compression and evaluated the interaction and strength effects of these defects during crack development under load. Mostafa Asadizadeh et al. [2] performed numerical simulations on cylindrical specimens with cracks under axial load and studied the effects of the inclination, length, and aperture of individual fractures on the mechanical behavior of the rock mass, crack initiation, and propagation. Jinhyun Choo et al. [3] studied the influence of size effects on the strength and crack propagation mode of gypsum samples with single and double defects through experiments and numerical simulations. Hadi Haeri et al. [4] studied an L-shaped notch specimen using the Brazilian test, biaxial test, and numerical simulation and revealed the influence of the distance between two L-shaped fractures, the crack length, and angle on the failure mode, crack type, and strength of the specimen. Mansour Sharafisafa et al. [5,6,7] used 3D printing technology, digital image correlation (DIC), and bond particle modeling (BPM) to study the failure patterns in rock-like disk specimens with pre-existing defects in different states (including filled and unfilled) through Brazilian and impact load tests. The failure mechanisms, crack types, and the effects of fillers were revealed. The above study investigated the propagation of cracks in opaque materials such as rocks and gypsum through indoor experiments and numerical simulations. Due to the opacity of the experimental material, it is not possible to observe the initiation and propagation of internal cracks. Therefore, there are certain difficulties in observing internal crack propagation and analyzing the mechanism. Wang Haijun et al. [8] used 3D-ILC technology to prepare glass specimens containing cracks and employed photoelastic testing techniques to visually demonstrate the distribution characteristics of the internal stress field in cracked rock specimens under the three-point bending test method. Hanzhang Li et al. [9] used the 3D-ILC method to create internal cracks in glass specimens. They conducted three-point bending tests on glass specimens with different crack depths to study the influence of pre-existing closed cracks on the initiation load and failure load. They also provided crack propagation patterns and mechanisms. Zhitao Zhang et al. [10] used 3D-ILC technology to create purely enclosed internal cracks in glass specimens, conducted UCS tests, and performed numerical simulations to discuss and analyze Type I, II, and III cracks. The aforementioned scholars took advantage of the relatively high brittleness of glass to conduct a series of studies on crack propagation. Due to the significantly greater brittleness of glass compared with rocks, it is difficult to observe distinct crack initiation and propagation before reaching the peak load during compression tests. In only a short period of time near the peak strength can the initiation and propagation of cracks be observed. Therefore, it is challenging to capture the entire process of crack initiation, propagation, and penetration, especially under compression loading conditions, using glass materials. Bankim Mahant et al. [11] used real-time microcomputer tomography (m-CT) to study the development of total pores, connected pores, non-connected pores, microfractures, and different pore network properties of sandstone under different uniaxial loading conditions (zero load, 25, 50, 75% of peak load, and peak failure load). E. E. Damaskinskaya et al. [12] used acoustic emission (AE) and CT techniques to study the formation characteristics of the main fractures, the multifractal characteristics of the pause of the acoustic emission event, and the form of the energy distribution function of the acoustic emission signal in granite under quasi-static uniaxial compression. Vikram Maji et al. [13] performed freeze–thaw experiments on limestone and sandstone and used micro-computed tomography (μ-CT) to visualize the growth, coalescence, and transition to macroscopic cracks of rocks during freeze–thaw cycles. Hamed Akhondzadeh et al. [14] investigated cryogenic liquid N2 fracturing of bituminous coal at the pore scale through 3D X-ray micro-computed tomography. The results of μ-CT showed that a fractured surface with large pore size was generated. Connections were established between otherwise isolated pores and micro-crevices, which increases the connectivity of the pore network. LI Zhaolin et al. [15,16] studied the cracking process of rock fractures by real-time CT scanning under true triaxial conditions, and the results showed that the intermediate principal stress played a decisive role in the direction of crack propagation in rocks. Baicun Yangdeng et al. [17] combined CT scanning technology with a uniaxial loading device to collect CT images at different deformation stages using the image threshold segmentation method in the compression process, and the evolution process of the damage inside the rock is given. According to the above research results, CT scanning technology has great advantages in studying the crack evolution law existing in real rock and other materials. However, it is not possible to create internal cracks of a specified shape, size, and location in materials from real rocks, and it is still necessary to use rock-like materials to study the initiation and propagation of internal cracks. Therefore, considering the difficulty of test methods and data processing and the complexity of the test device, it is of practical value to develop a transparent rock to study the evolution law of internal cracks. S. M. Torres et al. [18] used polymethyl methacrylate (PMMA) as a transparent rock substitute to study the internal impact propagation and crack growth of PMMA specimens subjected to uniaxial stresses in different numbers and directions. A. Bahmani et al. [19] performed a mixed-mode I+II fracture toughness test using a brittleness model polymer (PMMA). The performance of the asymmetrical edge-notched disc bending specimen was evaluated experimentally. S. Pirmohammad et al. [20] proposed a disc-bending specimen for determining the fracture toughness of brittle materials with Type I, I/II mixed, and Type II fractures and successfully carried out multiple fracture experiments on marble and PMMA materials under different loading modes. T. Zhou et al. [21] studied 3D-printed resin-based artificial rocks, investigating the effects of the shape and angle of pre-existing single and double fissures on the sample’s strength, deformation, and crack propagation speed. Bangxiang Li et al. [22] used transparent rock-like materials containing three-dimensional cracks as the research subject and proposed a PFC3D particle velocity trend line method. and investigated the initiation mechanism of pre-existing single and double cracks. Wang, Hongyu. Dyskin, Arcady et al. [23,24,25,26,27] conducted a series of uniaxial and biaxial compression experiments on a brittle transparent resin material and studied the initiation and propagation modes of internal cracks and surface cracks. The relationship between loading direction and crack propagation mode was also given. The above scholars developed different relatively transparent rock-like materials. Although the transparent rock-like materials are relatively uniform and different from real rocks, they have reference value for the mechanism research and extension mode research of the whole process of crack initiation, extension, and penetration in prefabricated cracks.

Although previous studies on crack propagation inside rock masses have achieved certain results, the research on surrounding rock of underground tunnels with internal defects mainly focuses on the extension mode of through cracks around holes and the influence of cracks on mechanical properties. In order to address the above problems, this paper develops a kind of transparent rock-like material on the basis of previous studies, analyzes the initiation and propagation of pre-existing cracks in transparent, brittle materials, and reveals its propagation mechanism. The results of this experiment are undoubtedly of great benefit to the study of the instability and failure mechanism in the surrounding rock in tunnels.

## 2. Materials and Methods

### 2.1. Selection of Similar Materials

The propagation of embedded three-dimensional cracks primarily occurs within the specimen, using transparent materials for experimentation, enabling direct observation of the initiation and propagation processes of new secondary cracks. After prolonged compatibility testing, this study ultimately selected unsaturated epoxy resin and amine curing agents as analogous materials to simulate brittle rocks. The main components of unsaturated epoxy resin include unsaturated epoxy resin, benzyl alcohol, and polyether amine. The material exhibited excellent brittle fracture characteristics under negative temperature conditions and maintained good transparency after curing.

### 2.2. Crack Parameters

**Figure 1:**
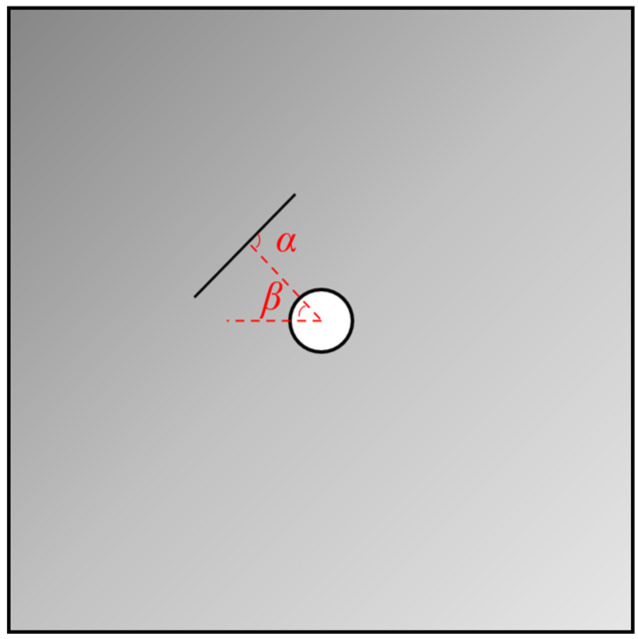
Front View of Pre-existing Crack. (α refers to the pre-existing crack inclination; The β angle represents the angle between the distance from the hole center to the center of the elliptical crack and the horizontal direction).

The experiment utilized PVC (Polyvinyl chloride) soft boards to simulate natural cracks within rock formations. PVC board has the characteristics of low hardness, easy plasticity, and good toughness, which can represent the original defect or notch in rocks. In the following, the original defects or notch in the rock were taken as the object of study, referred to as a pre-existing crack. The pre-existing crack shape was elliptical, with a major axis length of 24 mm, a minor axis length of 21 mm, and a thickness of 1 mm. The diameter of the circular hole at the center of the specimen was 10 mm. The distance from the center of the hole to the center of the crack was 22 mm. In Figure 1 below, the α angle refers to the angle between the distance from the hole center to the center of the elliptical crack and the pre-existing crack surface. The β angle represents the angle between the distance from the hole center to the center of the elliptical crack and the horizontal direction. The β angle was fixed at 30° in the design, while the α angle was set at four different angles: 0, 30, 60, and 90°. This variation was intended to investigate the impact of the pre-existing crack angle on crack propagation mechanisms and the compressive strength of the rock mass, as illustrated in Figure 1 below.

### 2.3. Specimen Preparation and Testing Method

**Figure 2:**
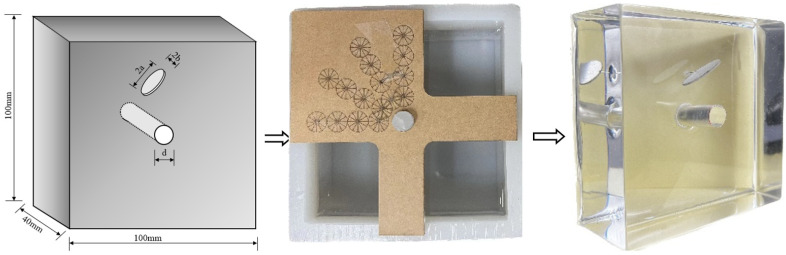
Schematic of Specimen Preparation with Pre-existing Cracks.

The dimensions of the uniaxial compression specimen for testing were 100 × 100 × 40 mm^3^ (length × height × width), as illustrated in Figure 2. The specimen preparation method is as follows: First, place the epoxy resin in a 40 °C environment and preheat it for 30 min to dilute the epoxy resin. Subsequently, uniformly mix the analogous material with a mixing ratio of epoxy resin, curing agent, and defoamer of 300:102:0.5. Stir the mixture thoroughly for at least 5 min, paying special attention to ensuring even stirring of the inner walls and bottom of the container until the stringy phenomenon is eliminated. Then, cure the mixture in a 30 °C environment for 72 h. After the curing period, place the specimen in a −60 °C environment and freeze it for 12 h. After the above treatment, the specimen essentially satisfies the two most important characteristics of rock materials: brittle characteristics and shear dilation characteristics under uniaxial compression conditions.

**Figure 3:**
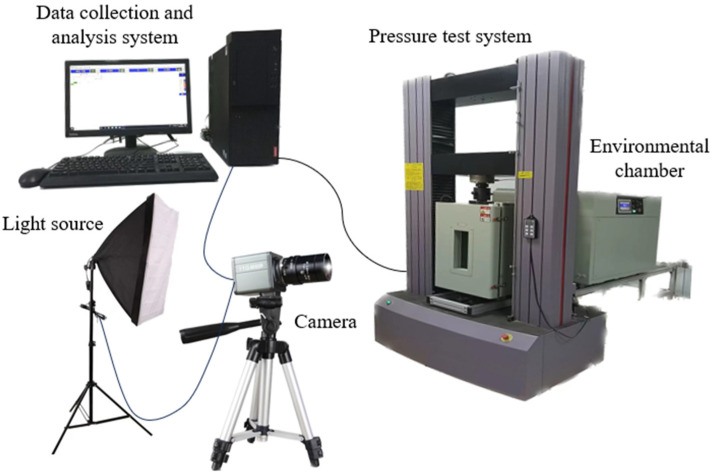
Uniaxial compression test equipment.

**Figure 4:**
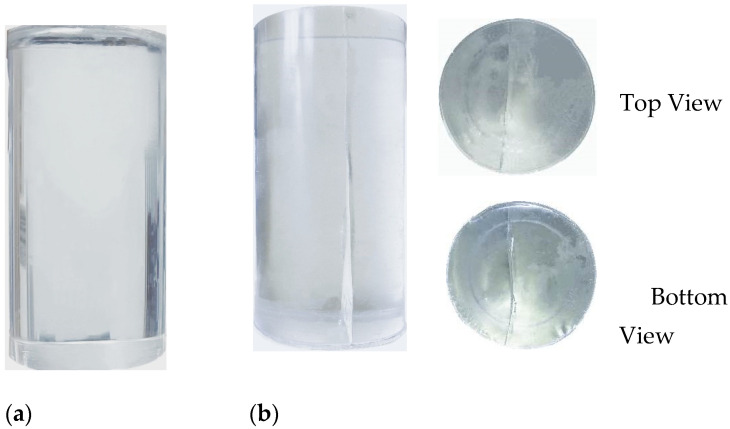
Results of the uniaxial compression test on the standard specimens: (**a**) Intact specimen; (**b**) Broken specimen.

The experiments were conducted on a multifunctional electronic control testing system that used the displacement control method for uniaxial compression testing with a control rate of 0.01 mm/s. According to the pre-existing crack inclination, the test specimens were divided into four groups, each consisting of five specimens with the same pre-existing crack inclination, and used a polytetrafluoroethylene (Teflon) gasket as the contact medium between the specimen and the test machine bearing platform. All the attribute parameters during the entire compression process were transmitted in real-time to the connected computer, and using a fill light and a high-definition camera with the same parameters, the specimen was photographed in a frontal and clockwise direction 60° away from the front, as shown in Figure 3 below. Throughout the experiment, the surface of the test specimen was regularly sprayed with an anti-fog agent and wiped to facilitate the documentation of the specimen’s failure process. This process aids in the post-experiment analysis of the extension and connectivity features of three-dimensional cracks. Table 1 shows the basic mechanical properties of frozen specimens tested at room temperature (25 ± 2 °C), such as uniaxial compressive strength (UCS), uniaxial tensile strength (UTS), and tensile compressive strength ratio (TCR). Figure 4 depicts the uniaxial compression failure of the intact specimen. The intact specimen was a cylindrical standard specimen with a diameter × height of 50 × 100 mm^2^.

## 3. The Fracture Test Results and Analysis

### 3.1. Evolutionary Mode of Three-Dimensional Cracks

#### 3.1.1. Cracks Inclination Angle α = 0° Situation

**Figure 5:**
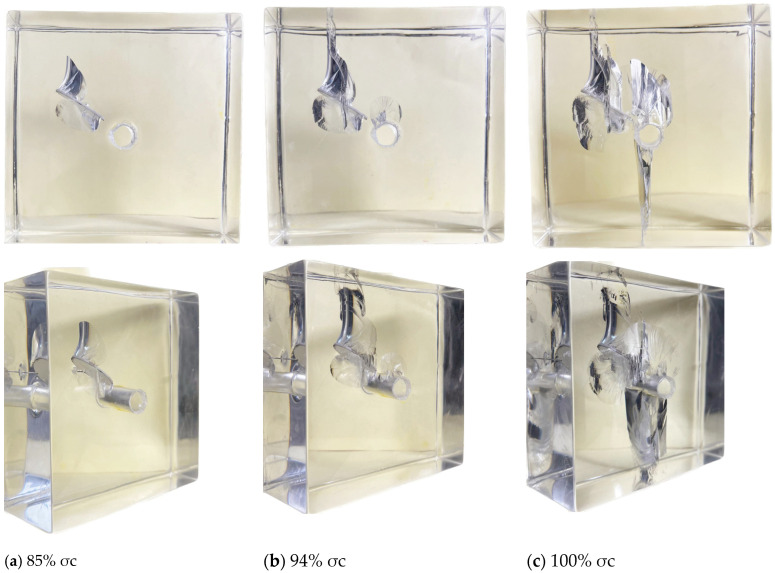
3D internal crack failure mode when the crack inclination angle α = 0°.

**Figure 6:**
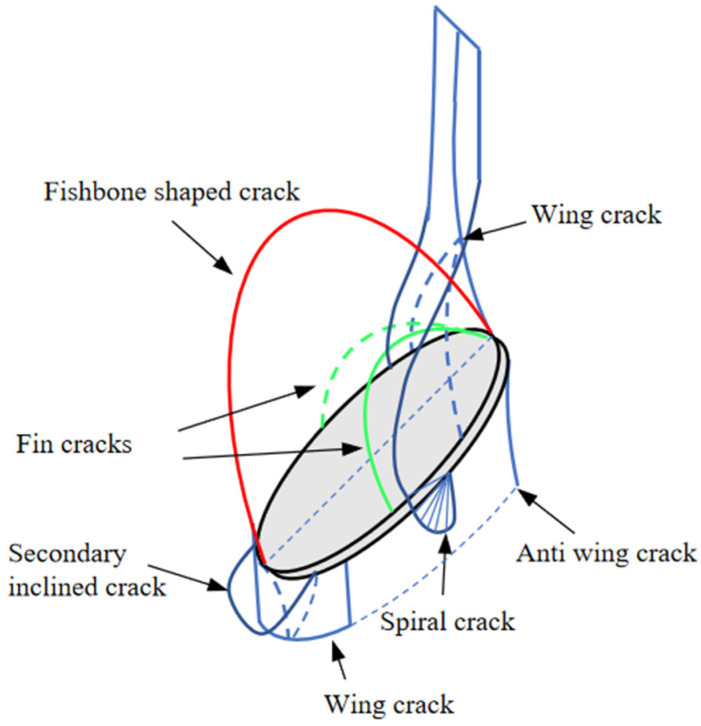
Reconstruction diagram of 3D crack propagation.

During uniaxial compression testing, it was observed that when σi (σi is the loading value of uniaxial load) increased to approximately 85% σc (σc is the peak strength of the specimen), the upper and lower tips of the pre-existing crack cracked almost simultaneously, resulting in an enveloping wing-shaped fracture. Compared with the enveloping crack at the upper tip of the pre-existing crack, the enveloping range and crack length were much smaller, as shown in Figure 5a. When the axial load σi increases to approximately 94% σc, cracking at the top of the cavity leads to fan-shaped secondary cracks, as shown in Figure 5b below. During this process, compared with the wing-shaped crack at the lower tip of the pre-existing crack, the crack at the upper tip of the pre-existing crack extends more noticeably upwards, reaching the top of the specimen. In contrast, the crack at the lower tip of the pre-existing crack shows a less significant downward propagation trend but extends in an enveloping manner along the edge of the elliptical pre-existing crack. Therefore, the wing-shaped crack at the lower tip exhibits a “self-limiting” effect in the vertical direction. This differs from the conclusion reached by Dyskin, A.V [28], where the “self-limiting” effect occurs after the crack extends to its ultimate length. As the axial load increased, secondary downward-inclined cracks appeared at the lower tip of the pre-existing crack, intersected with the cavity, and fishbone-shaped cracks appeared along the major axis direction on the upper surface of the pre-existing crack (as shown in Figure 6). This crack pattern is similar to Adams’s prediction model [29]. As the fishbone-shaped crack extended to the upper side of the circular hole, cracking appeared at the bottom of the hole simultaneously. Ultimately, the combination of wing-shaped cracks and cracks at the bottom of the hole led to the complete penetration failure of the specimen, as shown in Figure 5c below.

#### 3.1.2. Crack Inclination Angle α = 30° Situation

**Figure 7:**
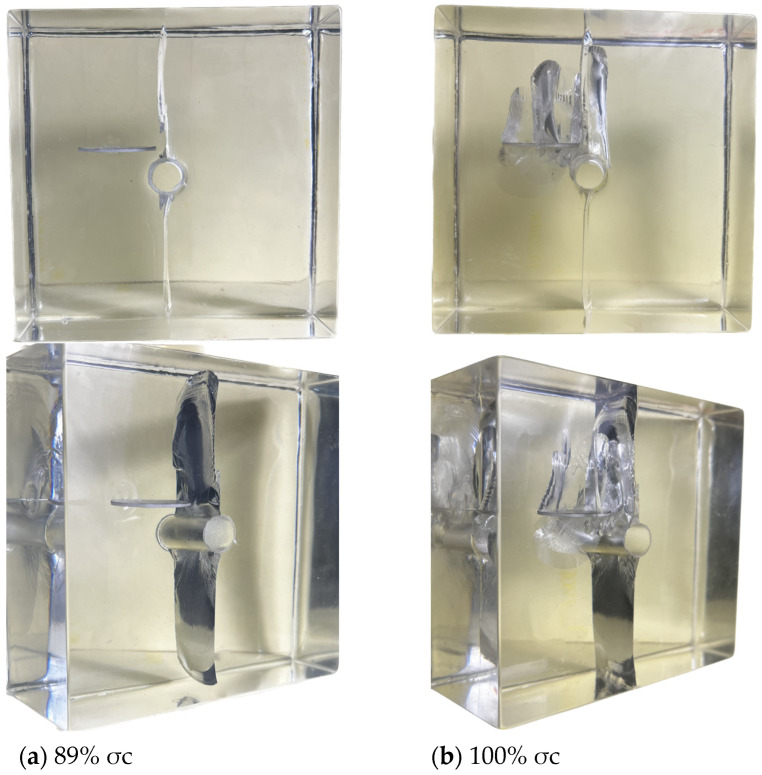
3D internal crack failure mode when the crack inclination angle α = 30°.

When the axial load σi increased to approximately 77% σc, microcracks first appeared on the surface of the pre-existing crack. Simultaneously, microcracks appeared at the right end of the pre-existing crack and extended in a downward diagonal trend towards the circular hole, nearing the top of the circular hole, as shown in Figure 7a below. When the axial load σi increased to approximately 89% σc, the top and bottom of the circular hole cracked almost simultaneously, rapidly extending to the bottom of the specimen. As the load further increased, cracks resembling a “cross-shaped intersection” appeared on the upper and lower surfaces of the pre-existing crack, and they extended in the vertical direction. From this, it can be inferred that under this inclination angle condition, the pre-existing crack does not lead to complete penetration failure of the specimen. The main reason for specimen failure is the cracking at the upper and lower parts of the hole, resulting in complete penetration failure of the specimen, as shown in Figure 7b below.

#### 3.1.3. Crack Inclination Angle α = 60° Situation

**Figure 8:**
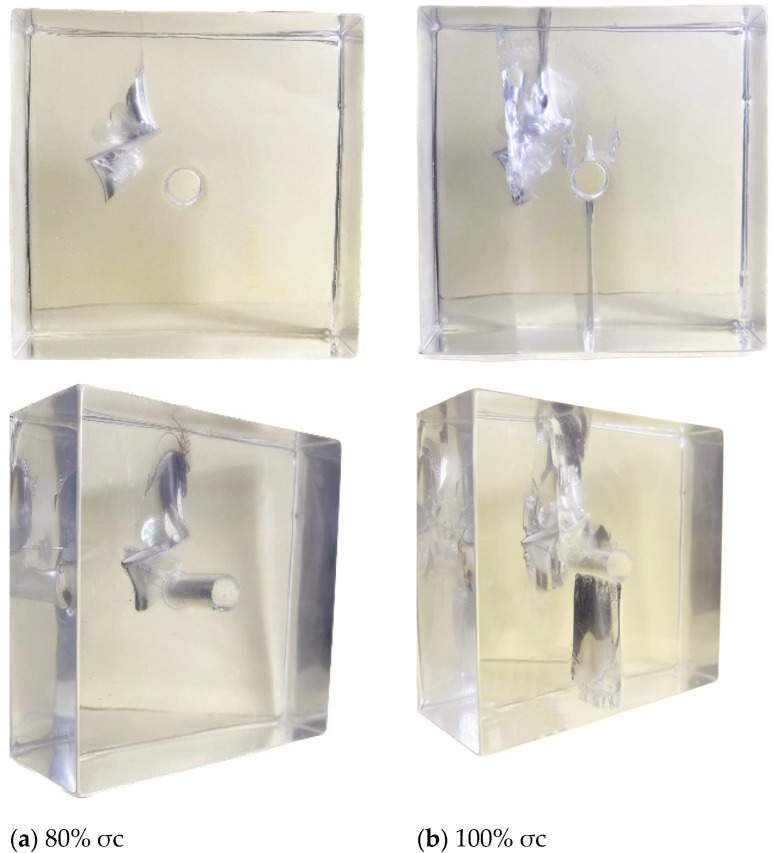
3D internal crack failure mode when the crack inclination angle α = 60°.

When the axial load σi increased to approximately 80% σc, cracks simultaneously appeared at the upper and lower tips of the pre-existing crack. The enveloping wing-shaped crack at the upper tip extended longer than the lower tip, and “fin-like” cracks appeared on the inner side of the enveloping wing-shaped crack, as shown in Figure 6 and Figure 8a. As the axial load continued to increase, the wing-shaped crack at the upper tip of the pre-existing crack continued to extend to the top of the specimen, while the crack at the lower tip barely extended, exhibiting a “self-limiting” effect in the vertical direction. At the same time, “fin-shaped” cracks on the upper and lower surfaces of the pre-existing cracks extended to the surface of the specimen, resulting in surface cracks. During this process, an enveloping anti-wing-shaped crack appeared on the lower side of the upper tip of the pre-existing crack (as shown in Figure 6 and Figure 8b) and penetrated through the circular hole. Simultaneously with the appearance of the above phenomena, cracking occurred at the top of the circular hole but did not penetrate to the top of the specimen. Cracks were also initiated at the bottom of the circular hole, extending to the bottom of the specimen. Ultimately, the combination of wing-shaped cracks and cracks at the bottom of the hole led to the complete penetration failure of the specimen, as shown in Figure 8b below.

#### 3.1.4. Crack Inclination Angle α = 90° Situation

**Figure 9:**
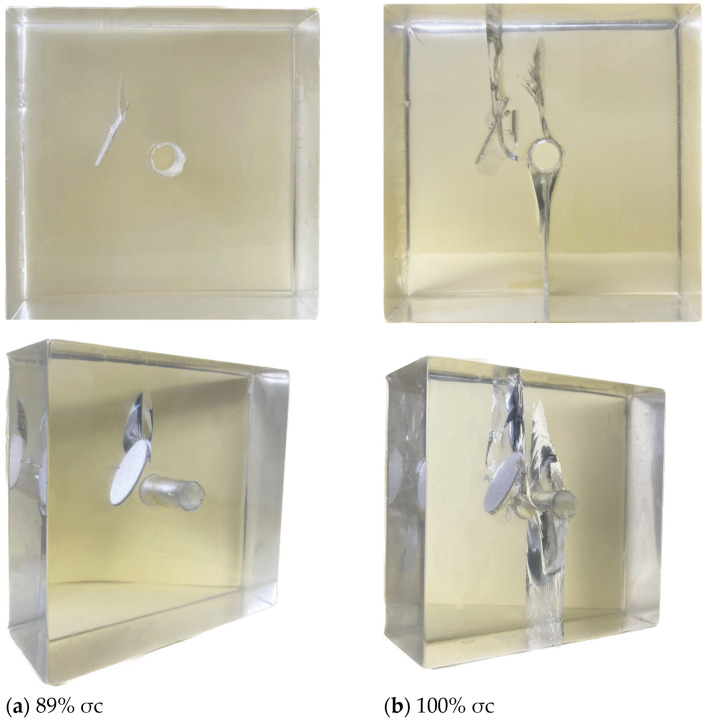
3D internal crack failure mode when the crack inclination angle α = 90°.

When the axial load σi reached approximately 89% σc, cracking initiated at the upper tip of the pre-existing crack, resulting in an enveloping wing-shaped crack, while no cracking occurred at the lower tip, as shown in Figure 9a. As the axial load further increased, cracking initiated at the lower tip of the pre-existing crack, with the wing-shaped crack at the upper tip also cracking. The crack area and length at the upper tip were significantly larger than those at the lower tip. The propagation direction of the wing cracks on both sides of the pre-existing crack moved away from the edge of the pre-existing crack and extended to the specimen surface. Simultaneously, the wing-shaped crack at the upper tip gradually extended downward on both sides of the specimen. At the same time, the extension direction underwent a significant turn towards the direction of the hole, ultimately penetrating through the circular hole. As the axial load σi continued to increase, cracking occurred almost simultaneously at the upper and lower parts of the hole. The crack at the lower part rapidly extended to the bottom of the specimen, while the crack at the upper part did not extend to the top of the specimen. During this process, an enveloping anti-wing-shaped crack appeared on the lower side of the upper tip of the pre-existing crack (as shown in Figure 6 and Figure 9b), almost connecting and penetrating through the circular hole. During this process, the growth trend of the surface crack generated by the wing-shaped crack at the upper tip of the pre-existing crack became more pronounced. However, the length of the wing-shaped crack at the lower tip did not increase, but the enveloping range expanded, exhibiting a “self-limiting” effect in the vertical direction. Ultimately, the combination of wing-shaped cracks and cracks at the bottom of the hole led to the complete penetration failure of the specimen, as shown in Figure 9b below.

### 3.2. Characteristics of Surface Cracks

**Figure 10:**
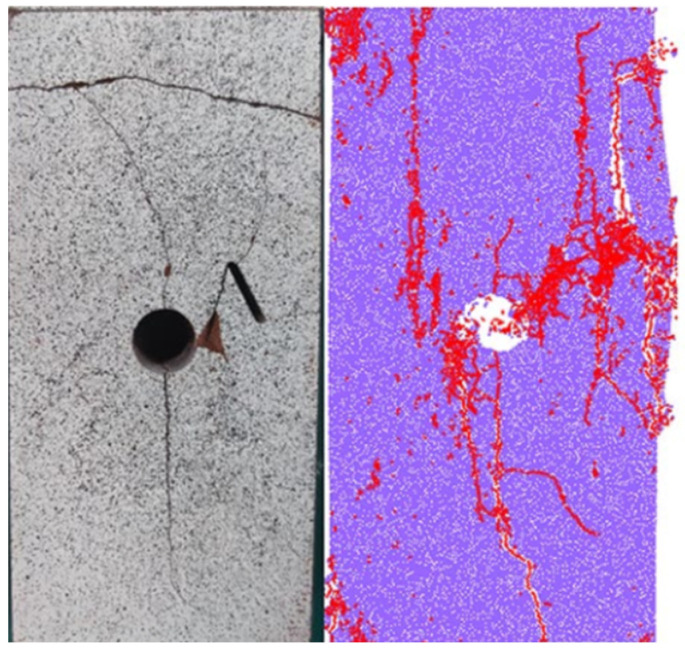
Failure diagrams of the experimental and numerical model at fissure angles of 30° [34].

**Figure 11:**
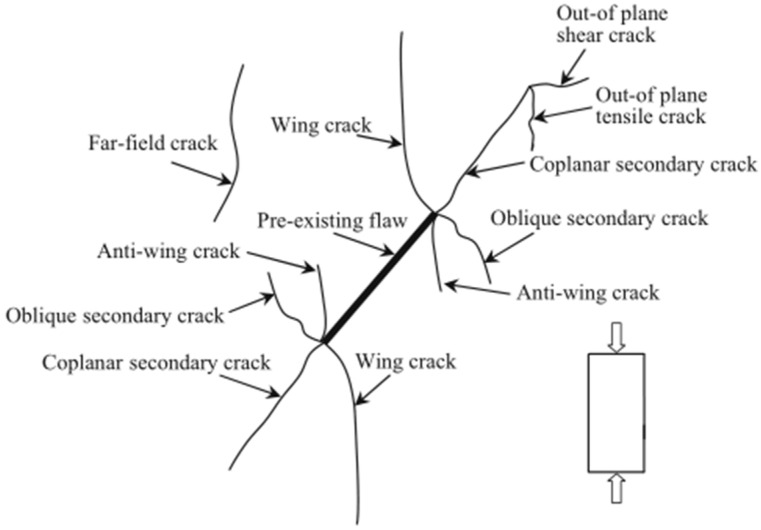
2D Crack types observed in rock specimens [35,36].

Many scholars have studied the propagation of penetrating cracks around holes, investigating the initiation, expansion, and penetration failure patterns under loading and conducting experimental and numerical simulations to understand the crack propagation modes and strength parameter regulations [30,31,32,33,34]. However, under the condition of internal three-dimensional cracks, the surface cracks observed on the specimen surface differ from the surface crack propagation observed in previous 2D studies [35,36], as shown in Figure 10 and Figure 11. Therefore, for opaque materials such as rocks, it is not feasible to analyze the failure characteristics of specimens with internal three-dimensional defects solely based on the observation of surface cracks.

**Figure 12:**
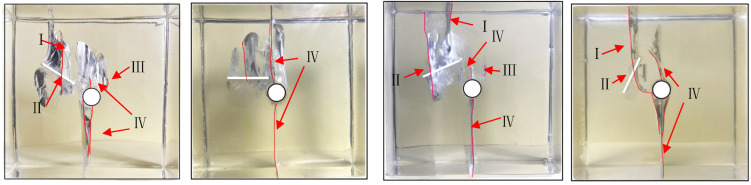
Surface Cracks of the specimen.

Based on the previous 2D experiments and numerical simulations, in the current experimental process, the initiation position of the cracks is consistent with previous experimental results, starting from the tip of the pre-existing cracks. However, unlike in the past, when the pre-existing crack tip initiated cracking, it did not immediately extend to the surface of the specimen. At the onset of pre-existing crack tip cracking, no cracks or macroscopic changes were observed on the specimen surface, yet significant cracking had already occurred within the specimen. Based on the results of this experiment, although establishing the spatial relationship between cracks caused by internal rock defects and surface cracks is challenging, it is evident that surface cracks on the specimen originate, grow, and extend due to internal crack initiation. The morphology and distribution of crack propagation closely correlate with geometric parameters, positions, and angles of pre-existing cracks. Mark the surface cracks on the specimen with red lines, as shown in Figure 12. Based on the experimental observations, the formation of surface cracks on the specimen can be summarized into the following four situations:

1. After the wing crack extends to a certain extent, it no longer follows the edge of the elliptical pre-existing crack and turns towards the surfaces on both sides of the specimen, resulting in surface cracks on the specimen with an almost vertical inclination, as shown in Figure 12I.
2. When the wing-shaped cracks extend to the vicinity of the short axis ends of the elliptical pre-existing crack, spiral cracks appear. These spiral cracks extend to the lower side of the pre-existing crack, and during the rotation process, they reach the specimen surface, causing surface cracks. As illustrated in Figure 12II.
3. As the axial load approaches the failure load, fishbone-like cracks and secondary cracks emerge, rapidly extending to the specimen surface. As illustrated in Figure 12III.
4. Surface cracks resulting from the top and bottom cracking of the hole are depicted in Figure 12IV.

## 4. Numerical Simulation

### 4.1. Establishment of the Particle Flow Model

**Figure 13:**
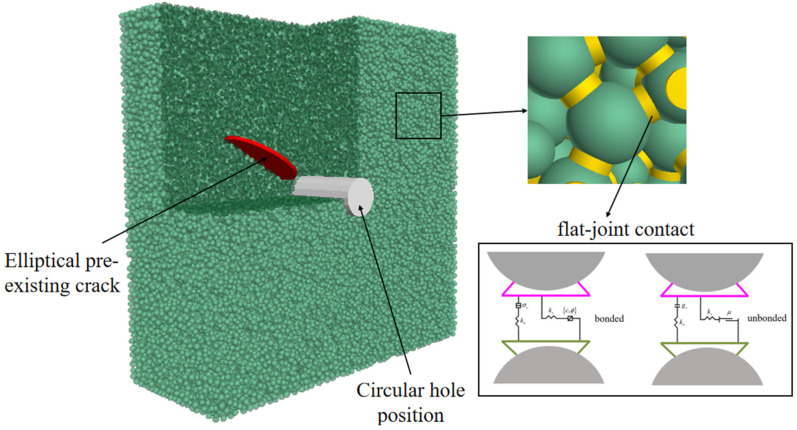
Numerical model and flat-joint contact.

Based on the dimensions of the specimen (The length, width and height were 100 × 40 × 100 mm^3^) and the geometric shape of the pre-existing crack, an elliptical pre-existing crack model was previously created in 3Dmax. Subsequently, using the Fish language, a circular hole at the center position was established. A numerical simulation specimen was then created using PFC3D particle flow analysis software. In addition to the predefined fissure and circular hole regions, particles were randomly distributed within the specimen dimensions, with the maximum particle diameter being smaller than the thickness of the pre-existing crack. Due to variations in the positions and radii of randomly generated particles inside the specimen, there were some discrepancies in the shape and size of the pre-existing crack, and the surface of the pre-existing crack was rough. Contact between particles was modeled using the flat joint model (FJM), while the contact between particles and walls utilized a linear contact model. The numerical model based on the FJM is illustrated in Figure 13. To replicate the relevant mechanical behavior of brittle resin materials, it was necessary to calibrate the microscopic parameters using macroscopic mechanical parameters obtained from laboratory tests. Displacement control was employed in the particle flow simulation test.

### 4.2. Determination of Microscopic Parameters

**Figure 14:**
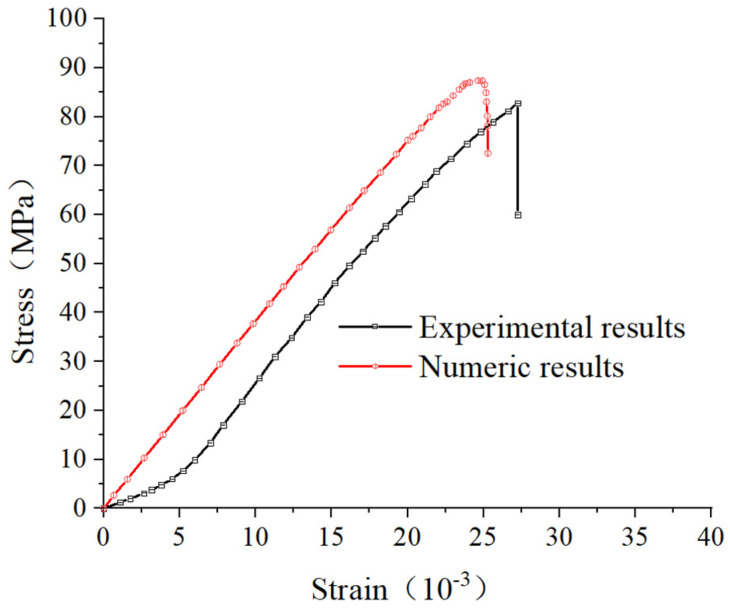
Comparison of numerical results and experimental results.

**Figure 15:**
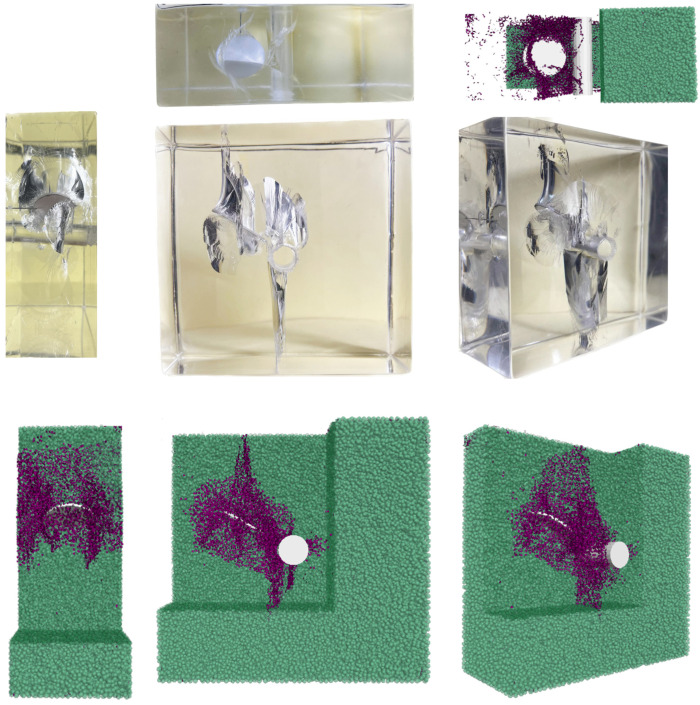
Comparison of numerical results and experimental results when α = 0°.

The “trial and error” method was employed to adjust the microscopic parameters of the particles iteratively. The numerical simulation results were compared with the laboratory experimental results, and the microscopic parameters were repeatedly adjusted to meet the requirements of the simulation analysis, ensuring consistency between the numerical simulation results and the laboratory experimental results. Finally, a final set of microscopic parameters that could reflect the characteristics of the laboratory specimen was determined, as shown in Table 2. The uniaxial compression stress–strain comparison curve between the laboratory test and the granular flow simulation specimen is shown in Figure 14. It can be observed that the stress–strain curve obtained using the macroscopic parameters from Table 2 fits well with the laboratory test results. This set of parameters can be used for the uniaxial compression testing of brittle resin-like rock materials, as shown in Figure 15 below.

### 4.3. Validation of the Numerical Model

To validate the effectiveness of the numerical model in simulating the three-dimensional cracking process, displacement-controlled uniaxial compression loading was applied to the numerical specimen. Under uniaxial compression conditions, with axial loads at peak strengths of 60, 80, and 100%, pre-existing cracks with different inclinations initiated and expanded. The simulation results are presented in Table 3. This table includes the front view and top view of the specimen, where the white ellipsoidal geometries and brown spots represent three-dimensional pre-existing cracks, holes, and fracture bonds, respectively. The concentration of fracture bonds in a specific region indicates the formation of macroscopic cracks. Upon comparing Figure 5, Figure 6, Figure 7 and Figure 8 with Table 3, it can be observed that the crack evolution pattern in the numerical simulation is essentially consistent with the laboratory experiment. This indicates that the FJM-based numerical model is capable of accurately simulating the cracking process of three-dimensional fissures.

## 5. Microscopic Mechanism of Crack Evolution

### 5.1. Displacement Trend Line Method

**Figure 16:**
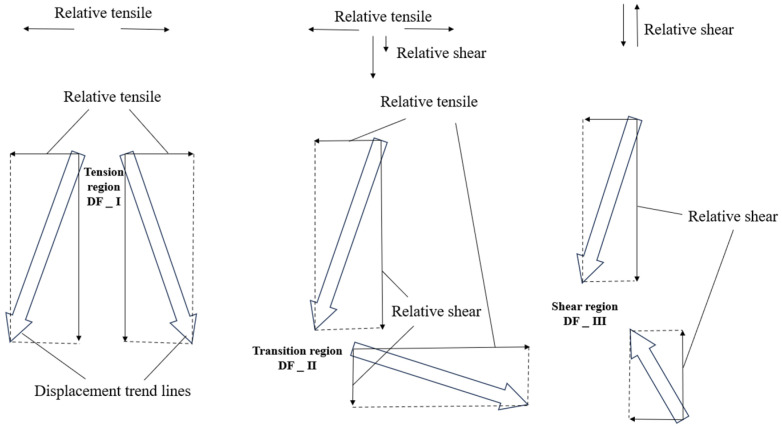
Three displacement field types defined by displacement trend lines.

In PFC3D, discrete particles cannot generate continuous stress and strain fields. Analytical methods in fracture mechanics are not suitable for application. To analyze the mechanisms of crack initiation and propagation, Zhang et al. [37] introduced a displacement vector trend line method, as shown in Figure 16. The relationship between particle displacement vector fields in Particle Flow Code and failure modes can elucidate the mechanisms of crack initiation for different types based on three primary conditions that might occur in numerical simulations. Hence, this principle is highly significant in the following discussion.

### 5.2. Crack Initiation and Propagation Patterns

**Figure 17:**
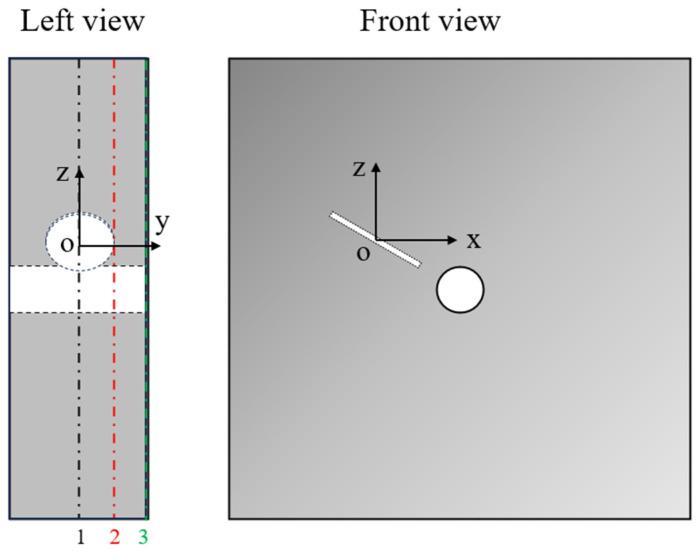
Specimen section position (1, 2, and 3 represent the three sections parallel to the XOZ surface and passing through the points (0,0,0), (0,10.5,0), and (0,20,0), respectively).

To investigate the initiation and propagation patterns of cracks around pre-existing cracks and circular holes, the specimens at Positions 1–3 were sectioned when subjected to axial loads at 90% of the peak strength. The crack initiation mechanism at the tip of the pre-existing crack, the rotation mechanism of wing-shaped cracks near the short axis of the pre-existing crack, and the mechanism of specimen surface failure caused by the internal crack propagation were studied at three sectional positions, as shown in Figure 17.

**Figure 18:**
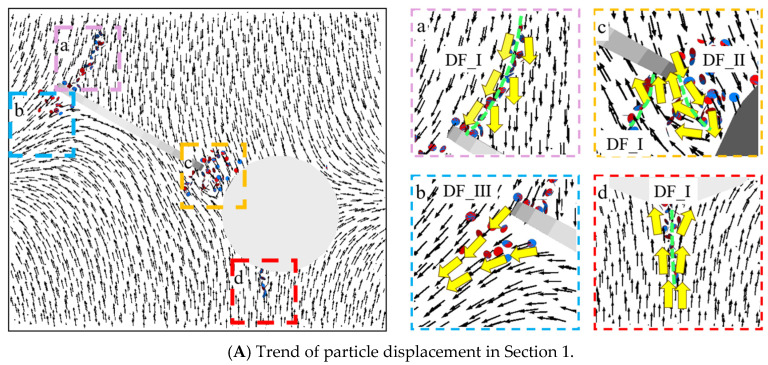

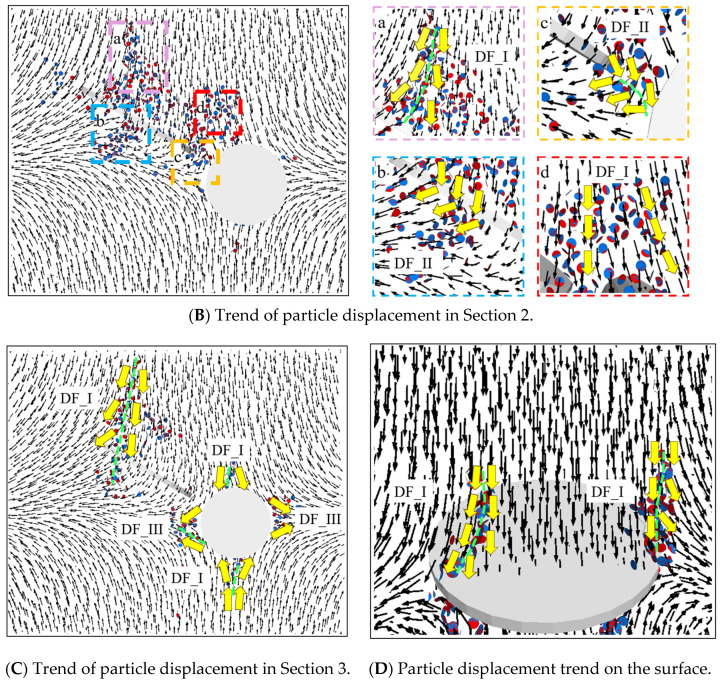
Particle displacement trend at α = 0°.

Based on Figure 18(Aa), it can be observed that at the tip of the pre-existing crack, there was a tendency for mutual deviation in particle displacement, leading to the formation of numerous concentrated fracture bonds, resulting in the formation of wing-shaped cracks. Thus, this region exhibited the DF_I relationship. As indicated by the particle displacement trend graph, the enveloping wing-shaped cracks formed due to the initiation at the tip of the pre-existing crack were tension-induced failure cracks. Based on Figure 18(Ab), it can be seen that the trend of the particle displacement on the lower left side of the pre-existing crack exhibited DF_III relationship, but only a few fracture keys appeared, and the relative displacement between the particles was small, without forming macroscopic cracks. Based on Figure 18(Ac), it can be seen that the particle displacement trend between the lower tip of the pre-existing crack and the circular hole exhibited two forms: the first type was characterized by DF_I relationship, forming wing-shaped tensile cracks; the second type exhibited DF_II relationship, forming secondary inclined tensile–shear mixed cracks (refer to Figure 6). Based on Figure 18(Ad), it can be seen that the particle displacement trend in the lower part of the hole area exhibited a DF_I relationship, and tensile cracks appeared.

Based on Figure 18(Ba), it can be seen that a large number of fracture bonds appeared near the short axis of the elliptical pre-existing crack, and the particle displacement trend in that area exhibited a DF_I relationship. The position and direction of the crack extension were consistent with the crack position and direction formed by the enclosed wing-shaped crack spiral at the short axis of the elliptical pre-existing crack in Figure 5. Therefore, the crack in that area was still formed by tensile action. Based on Figure 18(Bb), it can be seen that the particle displacement trend at the edge of the pre-existing crack exhibited a DF_II relationship controlled by shear action. Based on Figure 18(Bc), it can be seen that the particle displacement trend between the lower end of the pre-existing crack and the circular hole exhibited a DF_II relationship, forming secondary inclined tensile–shear mixed cracks (refer to Figure 6). Based on Figure 18(Bd), it can be seen that the particle displacement trend in the upper left area of the hole exhibited a DF_I relationship, with the appearance of tensile failure fracture keys, but no macroscopic crack was formed.

Based on Figure 18C, it was observed that the roughly vertical macroscopic crack formed on the specimen surface was a tensile crack, and the particle displacement trend exhibited a DF_I relationship. The upper and lower, left and right parts of the hole exhibited DF_I and DF_III relationships and, respectively, formed tensile cracks and secondary shear cracks.

Based on Figure 5 and Figure 6, it was found that “Fin cracks” appeared on the surface of the elliptical pre-existing crack. Combined with Figure 18D, it was revealed that the particle displacement trend at the two crack locations exhibited a DF_I relationship, and the “Fin cracks” at that location were controlled by tensile action.

**Figure 19:**
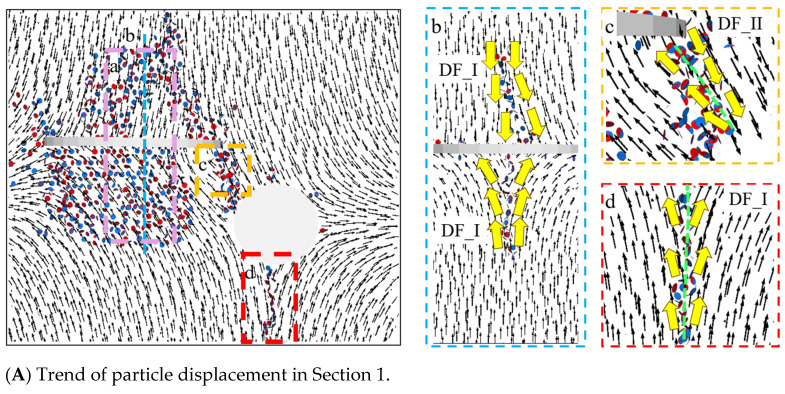

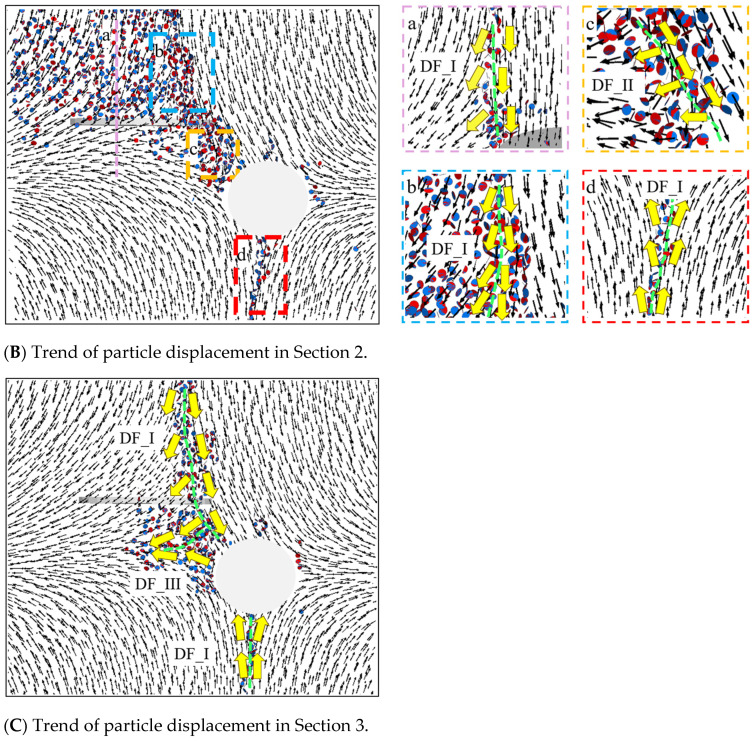
Particle displacement trend at α = 30°.

Based on Figure 19A, it can be observed that a large number of fracture bonds appeared on the upper and lower sides of the pre-existing crack in Region A. The particles on this surface only provide information in the X and Z directions, and the formation mechanism of these fracture keys cannot be determined based on the particle displacement trend on this surface alone. It is necessary to combine the particle information in the Y direction to determine the cracking mechanism. Therefore, at the central position of the pre-existing crack, a parallel cut was made along the Z-O-Y plane (Position B indicated by the blue dashed line in the figure below). Figure 19(Ab) reflects the particle displacement trend information in the Y direction in the upper and lower regions of the pre-existing crack. Further combined with Figure 19(Aa), it was found that the reason for the appearance of a large number of fracture bonds was that the particle displacement trend in the direction of the long axis of the elliptical pre-existing crack exhibited DF_I relationship, resulting in the appearance of numerous fracture bonds under tensile failure. Therefore, the cracks on the upper and lower sides of the pre-existing crack were both formed due to tensile failure. In Figure 19(Ac), the particle displacement trend between the pre-existing crack and the hole exhibited a DF_II relationship, and the cracks in this area were tensile–shear mixed cracks. In Figure 19(Ad), the particle displacement trend at the bottom of the hole exhibited a significant DF_I relationship, leading to the appearance of tensile cracks.

Based on Figure 19B, it can be observed that a large number of fracture keys appeared on the upper side of the pre-existing crack, and the particle displacement trend was not significant, making it impossible to determine the cause of the formation of the fracture keys. Therefore, particle displacement trend information in the Y direction is also required. A parallel cut was made along the Z-O-Y plane (position indicated by the pink dashed line a), resulting in Figure 19(Ba), which provides the particle displacement trend in the Y direction. Based on this figure, it was found that the particle displacement trend of the elliptical crack exhibited a significant DF_I relationship. The large number of fracture bonds that appeared on the upper side of the pre-existing crack were caused by tensile action, indicating that these cracks were formed due to tensile failure. Based on Figure 19(Bb), it can be observed that the particle displacement trend between the pre-existing crack and the hole exhibited a DF_II relationship, indicating that the cracks in this area were the result of tensile–shear mixed failure. In Figure 19(Bc), the particle displacement trend in the upper right corner of the pre-existing crack exhibited a significant DF_I relationship, leading to the appearance of tensile cracks. In Figure 19(Bd), the particle displacement trend at the bottom of the hole exhibited a significant DF_I relationship, resulting in the appearance of tensile cracks.

Based on Figure 19C, it was observed that on the upper left side of the hole, surface cracks formed by continuous concentrated fracture bonds appeared, and the particle displacement trend exhibited a DF_I relationship. According to Table 3, it was found that when the crack in the direction of the long axis of the elliptical crack was close to the end point of the long axis, a branching crack appeared, which was a tensile crack. Tiny cracks appeared in the left part of the hole, and the particle displacement trend in this crack area exhibited DF_II and DF_III relationships, controlled by tensile–shear mixed action.

**Figure 20:**
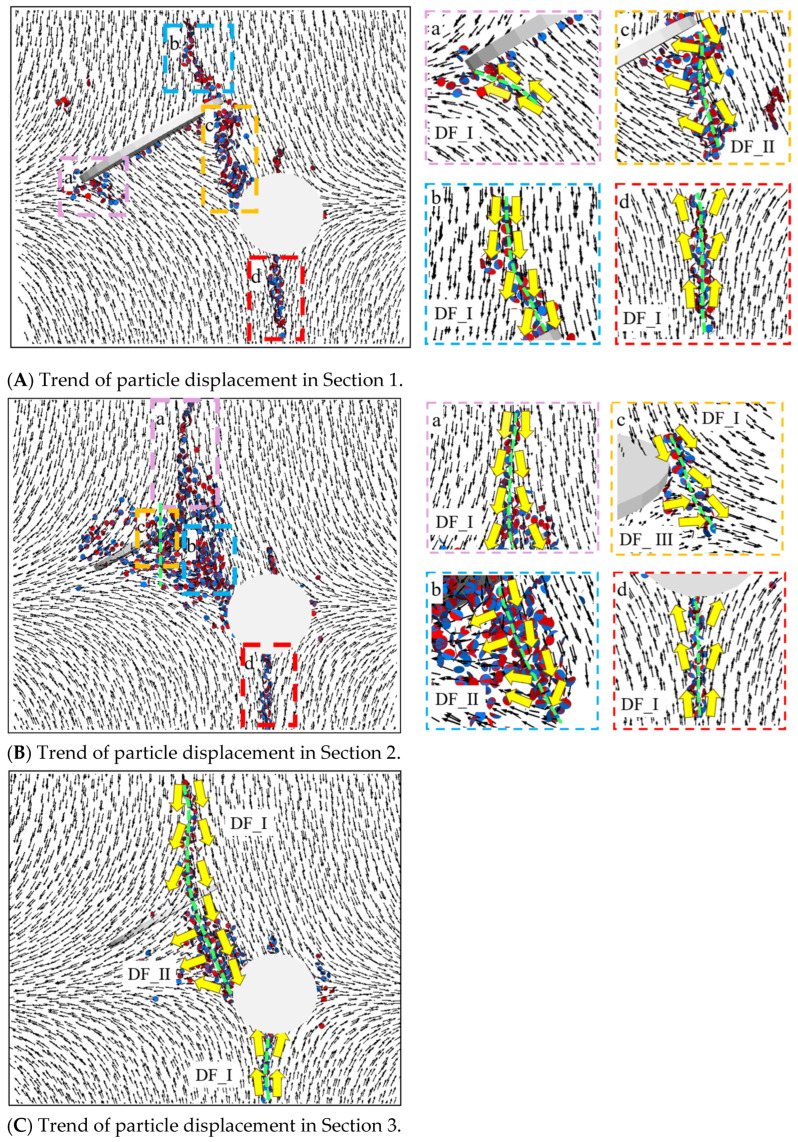
Particle displacement trend at α = 60°.

Based on Figure 20(Ab), it was observed that the particle displacement trend at the tip of the pre-existing crack exhibited a DF_I relationship, and the wing-shaped crack at this location was formed by tensile action. According to Figure 20(Aa), the wing-shaped crack was formed by tensile action, but the number of fracture bonds was significantly fewer than at the upper tip, and the crack extension length was not large. Based on Figure 20(Ac), it was observed that the particle displacement trend in the area from the lower end of the upper pre-existing crack to the hole exhibited a DF_II relationship, and the cracks in this area were formed by tensile–shear mixed action. According to Figure 20(Ad), the top and bottom of the hole exhibited a DF_I relationship, and the resulting fracture keys were influenced by tensile action, forming micro-cracks.

Based on Figure 20(Ba), it was observed that an enveloping wing-shaped crack formed at the upper tip of the pre-existing crack, extending along the edge of the elliptical pre-existing crack. The particle displacement trend at this location exhibited a DF_I relationship, and the wing-shaped crack on this section was formed by tensile action. The number of fracture bonds at the lower tip of the pre-existing crack was significantly fewer than at the upper tip, and no macroscopic cracks formed in this section. According to Figure 20(Bb), it was observed that the particle displacement trend in the area from the lower end of the upper tip of the pre-existing crack to the hole exhibited a DF_II relationship, and the cracks in this area were formed by tensile–shear mixed action. Based on Figure 20(Bc,d), it was observed that the fracture keys at the top and bottom of the hole were influenced by tensile action, forming micro-cracks.

Based on Figure 20C, it was observed that the enveloping wing-shaped crack extended to the endpoint of the short axis of the elliptical pre-existing crack. During this process, the wing-shaped crack was subjected to tensile action, and the particle displacement trend exhibited a DF_I relationship. The spiral crack appearing at the endpoint of the short axis of the pre-existing crack and the cracks below the pre-existing crack were influenced by tensile–shear mixed action, and the particle displacement trend exhibited a DF_II relationship. The fracture keys at the top and bottom of the hole were influenced by tensile action.

**Figure 21:**
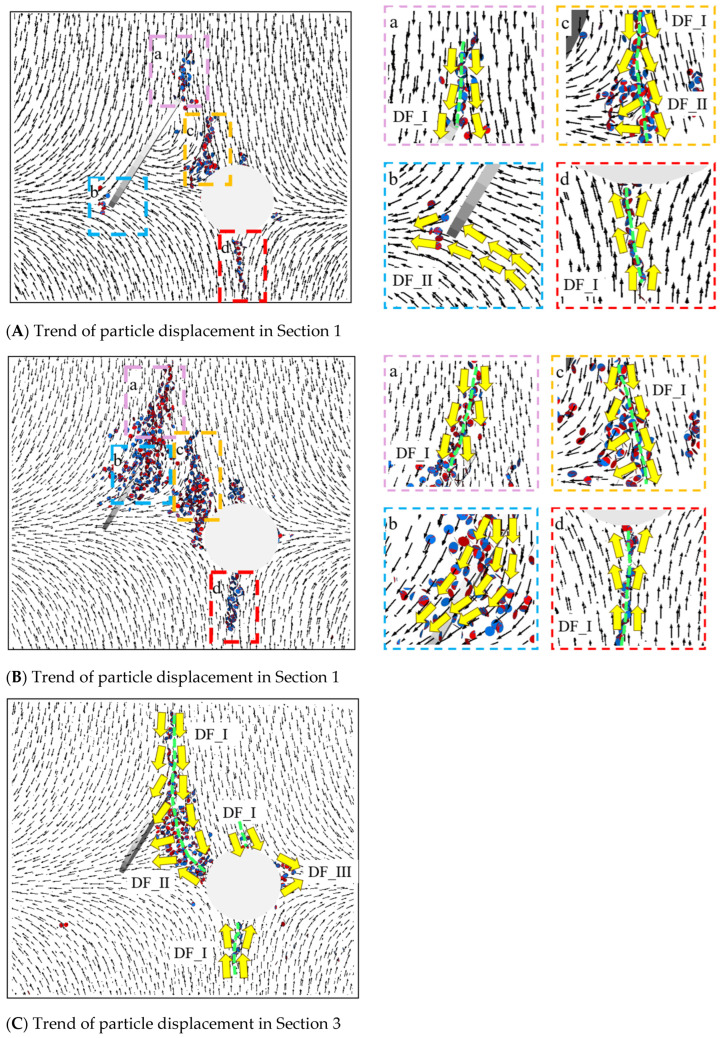
Particle displacement trend at α = 90°.

Based on Figure 21(Aa), it was observed that the particle displacement trend at the upper tip of the pre-existing crack exhibited a DF_I relationship, and the wing-shaped crack that appeared was due to tensile action. According to Figure 21(Ab), no obvious cracks appeared at the lower tip of the pre-existing crack. In comparison to the situation in Figure 20(Ac), in Figure 21(Ac), the particle displacement trend between the lower end of the upper pre-existing crack and the hole exhibited both DF_I and DF_II relationships, whereas in Figure 20(Ac), it exhibited a DF_II relationship. Based on Figure 21(Ad), it was observed that the particle displacement trend at the bottom of the hole exhibited a DF_I relationship controlled by tensile action.

Based on Figure 21(Ba), it was observed that the enveloping wing-shaped crack extended to the endpoint of the short axis of the elliptical pre-existing crack. During this process, the wing-shaped crack was a result of tensile action, and the particle displacement trend exhibited a DF_I relationship. According to Figure 21(Bb), there was a weak DF_II relationship at the ends of the short axis of the elliptical crack, but it was not significant. Based on Figure 21(Bc), it was observed that below the pre-existing crack, the cracks at the upper left corner of the hole were influenced by tensile and tensile–shear mixed action and the particle displacement trend exhibited both DF_I and DF_II relationships. According to Figure 21(Bd), the cracks at the bottom of the hole were due to tensile action.

Based on Figure 21C, it was observed that the enveloping wing-shaped crack extended to the endpoint of the short axis of the elliptical pre-existing crack. During this process, the wing-shaped crack was influenced by tensile action, and the particle displacement trend exhibited a DF_I relationship. The spiral crack appearing at the endpoint of the short axis of the pre-existing crack and the cracks below the pre-existing crack were influenced by tensile–shear mixed action, and the particle displacement trend exhibited a DF_II relationship. Tensile cracks appeared at the top and bottom of the hole.

Experimental and numerical simulations have been used to explain crack propagation under complex load paths [38,39]. According to the properties of the transparent, brittle material proposed in this paper, the specimen can be solidified in layers using layered pouring. Make the layer facet through the elliptical crack in the long-axis position. Taking advantage of the transparency of the specimen, the displacement field and stress field at the tip of the 3D pre-existing crack are obtained using digital image correlation (DIC) technology. Then, the crack propagation mechanism can be further compared and analyzed.

### 5.3. Microscopic Fracture Information Evolution Law

**Figure 22:**
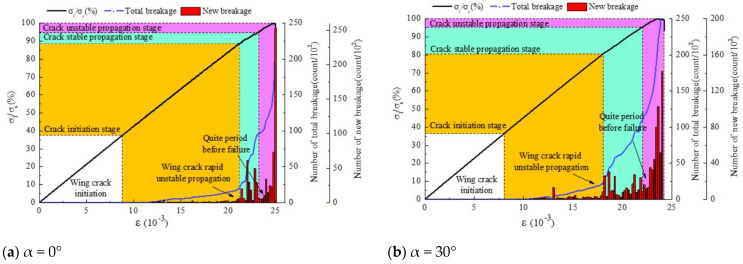

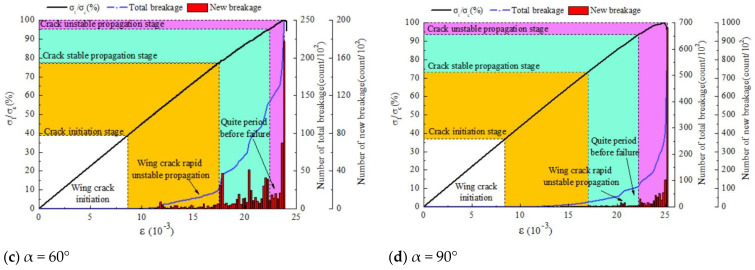
The variation in breakage quantity with increasing axial stress and strain.

Figure 22 shows the variation in breakage with an increase in axial load and deformation under different inclinations of the pre-existing cracks. The axial load σi is normalized using the peak strength σc. The red columns represent the amount of new breakage. The blue curve represents the cumulative crack number, and the black curve represents the stress–strain curve.

According to Table 4, it was found that the compaction stage ranged from 36.5% to 38.9% of the peak strength, and although the inclinations of the pre-existing cracks were different, the values of σi/σc differed by less than 2.5%. At this stage, no fracture keys appeared inside the specimens, and there was no crack information. The crack initiation stage ranged from 36.5 to 51.1% of the peak strength, and with an increase in the inclination of the pre-existing cracks, the incremental σi/σc required for the micro-crack stage increased. The stable crack propagation stage ranged from 6.3% to 20.4% of the peak strength, and with an increase in the inclination of the pre-existing cracks, the incremental σi/σc required for the micro-crack stage decreased. The unstable crack propagation stage ranged from 6.3% to 4.6% of the peak strength, and with an increase in the inclination of the pre-existing cracks, the incremental σi/σc required for the micro-crack stage showed a trend of initially decreasing and then increasing. However, the unstable crack propagation led to a relatively rapid penetration damage process, requiring stress increments of less than 6.3%.

## 6. Conclusions

Upon conducting indoor experiments and numerical simulations, this study evaluated the crack propagation patterns and micro-mechanisms of three-dimensional fractures around circular cavities and arrived at the following conclusions:

- A transparent, brittle material composed of epoxy resin, amine curing agent, and defoamer was developed, with a mixing ratio of 300:102:0.5. After low-temperature freezing, the tensile–compression ratio could reach 1/12 or more. This material could be used to study the initiation, propagation, and penetration damage patterns of internal cracks in brittle materials.
- Different inclination angles of the pre-existing cracks and different distances between the tips of the pre-existing cracks and the circular holes result in different crack initiation mechanisms being observed. When the inclination angle α of the prefabricated crack is 0°, tensile cracking occurs at the upper tip of the pre-existing crack, while wing-shaped tensile cracks and tensile–shear mixed cracks appear at the lower tip. When the inclination angle α of the prefabricated crack is 30°, no wing-shaped cracks appear, and the surface of the crack exhibits bifurcated tensile cracks only after the hole has cracked. At inclination angles of 60° and 90°, wing-shaped tensile cracks appear on the upper side of the pre-existing crack, while the anti-wing tensile crack and the anti-wing tensile–shear mixed crack appear on the lower side. Wing-shaped tensile cracks appear at the lower tip of the pre-existing crack, but with an increase in axial load, the extent of crack propagation is minimal, and ‘self-limiting’ phenomena occur in the vertical direction.
- As the crack extended to the surface of the specimen, the mechanism of crack propagation also changed. When the wing crack extended to both ends of the short axis of the pre-existing crack, a spiral crack appeared and extended to the underside of the pre-existing crack. During this process, the propagation mechanism transitioned from tensile cracking to tensile–shear mixed mode cracking. As the spiral crack extended to the surface of the specimen, generating surface cracks, the propagation mechanism transitioned from tensile–shear mixed mode to tensile cracking.
- The formation of surface cracks on the specimen can be summarized as being due to the following four situations:
  (1) After the wing crack extended to a certain extent, it no longer extended along the edge of the elliptical pre-existing crack, and the specimen’s surface turned towards both sides, extending to the specimen’s surface. The upward extension direction was almost perpendicular to the horizontal direction.
  (2) When the wing crack extended near the short axis of the elliptical pre-existing crack, spiral cracks appeared and extended downward to the surface of the specimen.
  (3) When the axial load approached the failure load, secondary cracks appeared and propagated instantaneously to the surface of the specimen.
  (4) Cracks are caused by cracking at the top and bottom of the hole.

The materials developed in this study are a continuum, which differs from the properties of natural rock materials, and the results of the study do not fully represent the internal crack propagation of real rocks. In the follow-up study, the addition of heterogeneous bodies and cryogenic bleed air increased the inhomogeneity of the transparent materials used in this test and further simulated the properties of natural pores and joints. This improvement has a certain guiding significance for exploring the internal crack propagation of brittle materials and real rocks.

## Figures and Tables

**Table 1:** Parameters of the new transparent resin.

Density (g/cm^3^)	Elastic Modulus (GPa)	UCS (MPa)	UTS (MPa)	TCR
1.1	3.5	116.67	9.10	12.82

**Table 2:** The microscopic parameters in the FJM.

Flat-Joint Model Parameters		Particle Parameters	
Particle and contact modulus, E∗ (GPa)	3.5	Minimum particle diameter, Rmin (mm)	0.8
Stiffness ratio of the particle and contact, kn/ks	2	Maximum to minimum particle diameter ratio, Rmax/Rmin	1.2
Tensile strength of the contact, σ_c_ (MPa)	30	Radius multiplier, *λ*	1.0
Cohesion of the contact, C (MPa)	33	Particle density, *ρ* (g/cm^3^)	2.7
Friction angle of the contact, φ (°)	30	Friction coefficient, μ	0.5
Number of elements in the radial direction, Nr	2	-	-
Number of elements in the circumferential direction, Na	4	-	-

**Table 3:** Numerical simulation results.

Stage	View	UCS 60%	UCS 80%	UCS 100%
α = 0°	Top view	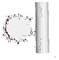	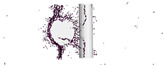	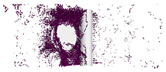
	Front view	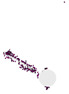	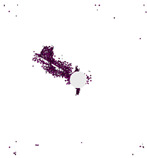	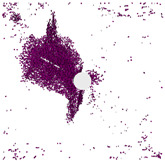
α = 30°	Top view	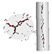	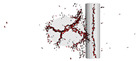	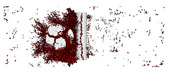
	Front view	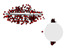	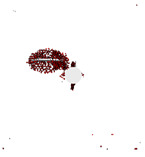	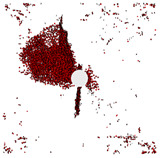
α = 60°	Top view	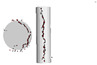	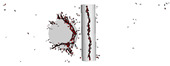	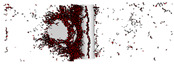
	Front view	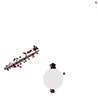	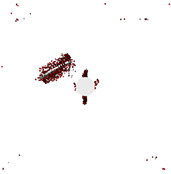	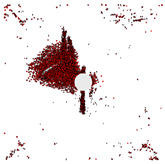
α = 90°	Top view	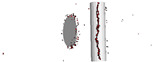	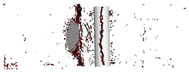	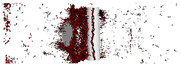
	Front view	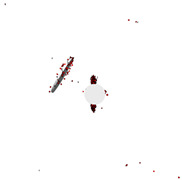	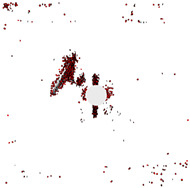	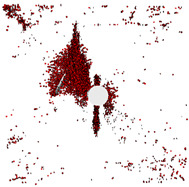

**Table 4:** Stress increment required for crack propagation.

Pre-Existing Crack Inclination α(°)	Increment of σ_i_/σ_c_ (%)			
	Compaction Stage	Crack Initiation Stage	Crack Stable Propagation Stage	Crack Unstable Propagation Stage
0	36.8	36.5	20.4	6.3
30	38.9	38.4	17.9	4.8
60	36.5	44.0	14.9	4.6
90	37.6	51.1	6.3	5.0

## Data Availability

The data used to support the findings of this study are included within the article.
